# Intracranial Hemorrhage Detection Using Parallel Deep Convolutional Models and Boosting Mechanism

**DOI:** 10.3390/diagnostics13040652

**Published:** 2023-02-09

**Authors:** Muhammad Asif, Munam Ali Shah, Hasan Ali Khattak, Shafaq Mussadiq, Ejaz Ahmed, Emad Abouel Nasr, Hafiz Tayyab Rauf

**Affiliations:** 1 Department of Computer Science, COMSATS University Islamabad, Islamabad 44000, Pakistan; 2 School of Electrical Engineering and Computer Science (SEECS), National University of Sciences and Technology (NUST), Islamabad 44500, Pakistan; 3 Institute of Computing, Kohat University of Science & Technology, Kohat 26000, Pakistan; 4 Computer Science Department, National University of Computer and Emerging Sciences (NUCES-FAST), Islamabad 44000, Pakistan; 5 Industrial Engineering Department, College of Engineering, King Saud University, Riyadh 11421, Saudi Arabia; 6 Centre for Smart Systems, AI and Cybersecurity, Staffordshire University, Stoke-on-Trent ST4 2DE, UK

**Keywords:** intracranial hemorrhage, computed tomography, light gradient boosting machine, support vector machine, convolutional neural networks

## Abstract

Intracranial hemorrhage (ICH) can lead to death or disability, which requires immediate action from radiologists. Due to the heavy workload, less experienced staff, and the complexity of subtle hemorrhages, a more intelligent and automated system is necessary to detect ICH. In literature, many artificial-intelligence-based methods are proposed. However, they are less accurate for ICH detection and subtype classification. Therefore, in this paper, we present a new methodology to improve the detection and subtype classification of ICH based on two parallel paths and a boosting technique. The first path employs the architecture of ResNet101-V2 to extract potential features from windowed slices, whereas Inception-V4 captures significant spatial information in the second path. Afterwards, the detection and subtype classification of ICH is performed by the light gradient boosting machine (LGBM) using the outputs of ResNet101-V2 and Inception-V4. Thus, the combined solution, known as ResNet101-V2, Inception-V4, and LGBM (Res-Inc-LGBM), is trained and tested over the brain computed tomography (CT) scans of CQ500 and Radiological Society of North America (RSNA) datasets. The experimental results state that the proposed solution efficiently obtains 97.7% accuracy, 96.5% sensitivity, and 97.4% F1 score using the RSNA dataset. Moreover, the proposed Res-Inc-LGBM outperforms the standard benchmarks for the detection and subtype classification of ICH regarding the accuracy, sensitivity, and F1 score. The results prove the significance of the proposed solution for its real-time application.

## 1. Introduction

Intracranial hemorrhage (ICH) occurs within the cranium due to a traumatic brain injury, tumor, stress, vascular abnormality, arteriovenous malformations, and smoking [1,2,3]. One of the major concerns of ICH is the high death rate of about 35% to 52% in the first 30 days [4,5]. Other concerns such as disability, epilepsy, vascular issues, blood clotting, loss of memory, and vision are also faced by the survivors [6,7]. Therefore, a rapid and accurate mechanism is required to give medical treatment at the initial level to reduce the high mortality rate of ICH. Primarily, computed tomography (CT) scans are used by radiologists to locate the ICH region and type manually. These scans are based on adjacent slices examined by the radiologist to identify the region and pattern of hemorrhage [8]. The ICH region can be intra-axial or extra-axial. The intra-axial represents the bleeding inside brain tissues, whereas the bleeding inside of cranial but outside of the brain tissues is known as extra-axial. The first region covers two types: intraparenchymal hemorrhage (IPH) and intraventricular hemorrhage (IVH), whereas subarachnoid hemorrhage (SAH), subdural hemorrhage (SDH) and epidural hemorrhage (EDH) are covered by the second region [9,10].

The primary way of ICH detection and subtype classification is to physically analyze the CT images of the human head by radiologists. However, due to subtle complexities, overloaded burden, and the time-consuming task of ICH detection, the expertise of radiologists is required to rapidly and accurately detect the ICH region and its cause [9,11]. Another issue that leads to a high death rate is the unavailability of expert radiologists at an emergency time in many developing countries [11]. Therefore, the junior radiologists analyze the CT scans and detect the cause and region of ICH. Although there is a difference in the detection of ICH by expert radiologists than the juniors, which affects on the treatment and medication [9,12]. Therefore, to deal with the large dataset, streamline workflow, timely availability of the detection system, and improve the ICH detection accuracy, an artificial-intelligence-based automated mechanism is required [7,12,13]. Recently, many studies have been proposed to automate ICH detection and subtype characterization, classified into machine learning and deep-learning-based solutions.

Machine-learning-based solutions for identifying ICH have risen significantly in the past few years. ICH has different types in which subarachnoid hemorrhage (SAH) is one of them. The authors in [14,15] propose a decision tree (DT) and random forest 50 (RF)-based model using the clinical data of patients to detect the SAH.

Likewise, Ramos et al. [16] employ four efficient machine learning models, known as DT, RF, support vector machine (SVM), and multilayer perceptron (MLP), to improve the detection of SAH using the laboratory dataset of patients. Another study [17] proposes a hybrid of deep learning and machine learning model where a deep convolutional neural network (CNN) is used to extract features and linear SVM to detect ICH using head CT scans.

Raima et al. [11] detect the presence of ICH using SVM. Afterwards, U-Net segmentation is performed to locate and classify the ICH region. The researchers in [18] show the performance comparison of six machine learning models for the spontaneous detection of ICH. The machine above learning algorithms improve ICH detection and subtype classification results. However, the presented solutions require significant manual data preprocessing, parameters optimization, and feature engineering. Furthermore, these solutions become less efficient and lead to overfitting as the size of the dataset increases [8,9,13]. Thus, with the increasing number of cases globally, data size is increasing, which requires a more effective approach, known as deep learning, to overcome the shortcomings.

Deep learning approaches are efficient in handling large datasets and automating the feature extraction from brain CT slices, attracting the research community’s attention. Sage et al. [19] employ two parallel paths based on ResNet50 to capture the potential features from the head CT scans. These features are forwarded as input to the random forest (RF) and SVM for the detection of ICH. The researchers in [20] propose a deep CNN model based on five convolutional layers to extract features and two dense layers for the identification of ICH. Grewal et al. [21] perform the ICH detection using the 3D CT images where a baseline architecture of DenseNet is used along with the recurrent neural network (RNN). Wu et al. [8] employ the deep CNN model, known as EfficientNet-B0, in two parallel paths to obtain both brain tissue and spatial-based features. Afterwards, the ensemble mechanism is applied to the outputs to perform the ICH detection. In [12], Lee et al. execute four deep learning models, VGG16, Inception-ResNetV2, Inception-V3, and ResNet50, where CNN is used as the baseline architecture. The models are combined to form an ensemble mechanism for improving the detection of ICH. Mansour et al. [22] perform the segmentation to identify the affected regions using a segmentation technique known as Graph-Cut.

Furthermore, the Capsule network is used for feature extraction and fuzzy deep neural network to detect and characterize ICH. Another work in [23] demonstrates the importance of brain windows setting to improve the detection of ICH. The authors attach a window estimator module with the deep CNN model to automate the estimation of brain windows setting.

Anupama et al. [24] locate the ICH regions using the Grab-Cut segmentation mechanism. Moreover, a synergic deep learning concept is used for feature extraction and subtype classification of ICH using head CT images. Wang et al. [2] and Ye et al. [25] identify ICH and its subtypes through a hybrid of CNN- and RNN-based models using 3D CT images. The intention behind this is to extract important features using CNN. Then, RNN performs sequential learning and detection of ICH following a similar approach [5,26,27] where a CNN-based architecture is used to capture features from 3D brain CT images. Then, the long short-term memory (LSTM) model uses the high-level features provided by CNN and performs correlation analysis at slices to identify the ICH and its subtypes. In [28], a faster R-CNN-based architecture is proposed to localize the ICH region and its type categorization using brain CT images.

Lee et al. [29] apply a conventional artificial neural network (ANN) model to demonstrate its acceptability over the CNN model regarding detection and subtype classification of ICH. The aforementioned deep learning solutions significantly rise to ICH detection and its subtype classification performance. However, some of the answers are based on 3D CT images, which require extra time and memory for model training due to a high number of parameters [21,25,28]. Furthermore, it is important to capture spatial features from adjacent CT slices in a similar way that have a significant impact on the ICH detection performance, which is not covered by the studies [5,11,21,25,27]. Few of the studies [14,17,19] have employed machine learning techniques such as SVM, DT, and RF for the ICH detection using the CNN-based features, which are less effective for large dataset and prone to overfitting.

To address the limitations mentioned above, we propose a new deep learning and machine learning-based hybrid model for ICH detection and subtype classification. The proposed solution employs two deep learning models, ResNet101-V2 and Inception-V4, in parallel ways to extract the potential features from brain CT slices. The architecture of these solutions consists of CNN. The ResNet101-V2 captures intensity-related elements from windowed CT slices, whereas the spatial features from adjacent pieces are obtained through the Inception-V4. The prime intention behind extracting spatial and intensity features are to mimic radiologists’ real-world diagnostic processes. Afterwards, a more efficient and fast technique, a light gradient boosting machine (LGBM), is applied to the extracted features by windowed and spatial features to detect the ICH and its subtypes. Therefore, the proposed solution is a combined ResNet101-V2, Inception-V4, and LGBM (Res-Inc-LGBM) based model for ICH detection. To evaluate the performance of the proposed Res-Inc-LGBM, extensive experimentation is performed using the dataset of intracranial hemorrhage detection challenge (IHDC) provided by the Radiological Society of North America (RSNA). Moreover, the proposed solution is tested on the CQ500 dataset to analyze its generalization.

## 2. Materials and Methods

**Figure 1:**
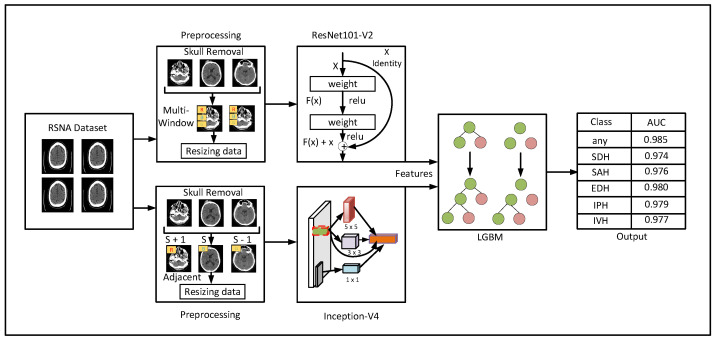
Systematic view of the proposed methodology for detection and subtype classification of ICH.

In this section, we present a detailed description of the proposed methodology for detection and subtypes classification of ICH, as shown in Figure 1. The proposed method consists of three major phases: preprocessing, feature extraction, and ICH detection. Thereafter, in the preprocessing phase, noncontrast brain CT images are used as input data. This phase includes techniques such as skull removal and multiwindowing to enhance ICH detection. Afterwards, the feature extraction phase utilizes two excellent deep learning models to capture intensity-based and spatial features from the given CT scans, as shown in Figure 1. Then, an LGBM model detects the ICH and its subtypes in the third phase using the extracted features from the previous phase. In this regard, the proposed method efficiently overcomes the shortcomings of the existing models for ICH detection and subtype classification. The description of each phase is given in the following subsections.

### 2.1. Data Preprocessing

The proposed mechanism for the identification and characterization of ICH requires the preprocessing of input to smooth the training process and reduce the training time of the model. Therefore, we apply preprocessing techniques such as skull removal, windowing to capture brain and bone tissues, and data augmentation.

#### 2.1.1. Dataset

**Figure 2:**
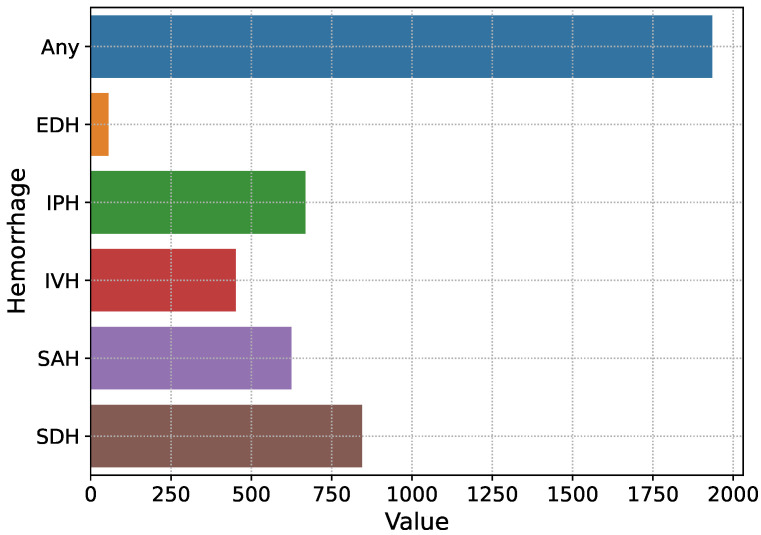
Overview of subtypes of ICH.

**Figure 3:**
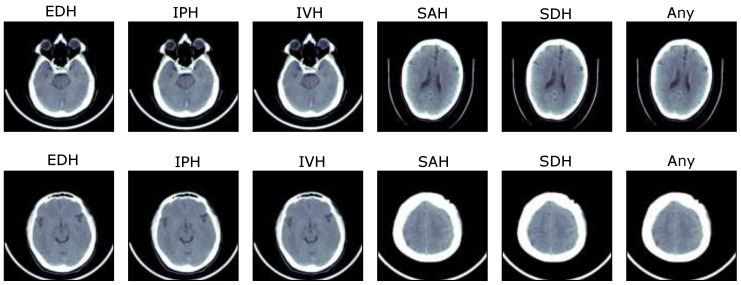
Representation of subtypes of ICH.

The proposed solution is trained and evaluated over the brain hemorrhage data of ICH patients, known as IHDC, which was made available in 2019 by the RSNA [2,30,31]. The dataset is collected from three universities, which is further evaluated by about 60 radiologists of RSNA and made available to develop automated solutions for the detection and subtype classification of ICH [2]. The dataset consists of noncontrast head CT slices in digital imaging and communications in medicine (DICOM) annotated with IPH, IVH, SAH, SDH, and EDH. The slices that contain no hemorrhage or more than one are annotated as ‘Any’. The selected dataset for the training and validation of the proposed Res-Inc-LGBM solution includes information on 13,334 patients with different types of brain hemorrhages. The total number of brain CT images containing hemorrhage is 4579, as shown in Figure 2. The general representation of subtypes of ICH is shown in Figure 3. In addition, another dataset is employed to analyze the proposed solution’s generalisation ability, known as CQ500 [32]. It consists of head CT scans collected from different radiological centres, annotated by expert radiologists. Furthermore, detailed information about both datasets is given in Table 1.

#### 2.1.2. Multiwindowing and Adjacent Slicing

Here, we elaborate on the two types of preprocessing, windowing and adjacent slicing. Before that, we apply the skull removal process that improves ICH detection by specifically focusing on the brain region. Moreover, it improves the model’s training without focusing on the skull region. Therefore, Otsu’s morphological method removes the skulls of head CT images [33,34]. Afterwards, we applied the windowing strategy, known as Hounsfield units, to capture the intensity-based features. This strategy improves the contrast level of head CT scans through window width and level to capture the bone, brain, and subdural tissues [2,35].

**Figure 4:**
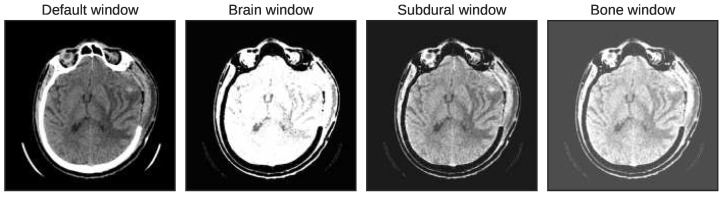
Representation of three intensity windows.

Moreover, in the actual application, the radiologists also adjust the windows of brain CT slices to improve the contrast for better locating the lesions. The prime intention is to focus on the specific tissues to discover the hidden complexities of ICH [8]. In this paper, we have applied three intensity windows, termed brain window, bone window, and subdural window, to improve the visualization of brain tissues, skull lesions, and soft tissues, as shown in Figure 4. The first window focuses on the brain tissues where window width and level are defined as 80 and 40, respectively. Afterwards, the window width of 380 and window level 40 are set to focus on the bone tissues. The third window, the subdural window, is obtained by setting the window level to 80 and the width to 200, which captures the subdural hematomas. The three intensity windows are combined to form a single image with three channels.

The other type of preprocessing, the adjacent slicing mechanism, helps the proposed model to focus on the adjacent slices for locating the bleeding regions. Furthermore, this mechanism mimics the real-time diagnostic process of radiologists that use adjacent slices to find the subtle bleeding spots and improve the detection process Wu et al. [8,12,36]. Therefore, to capture the spatial features, a slice interpolation, also known as an adjacent slicing mechanism, is utilized to improve the ICH detection process. In this mechanism, two adjacent slices and the centre slice are concatenated to form a single image with three channels. After applying windowing and adjacent slices, an image augmentation procedure is used to enhance the generalization ability of the proposed solution. In addition, it resolves the overfitting issue. The augmentation method includes scaling, flipping, translating, zooming, and shearing range to get different representations. Afterwards, the slices are rescaled to 224 × 244 to match the model’s input representation because the original CT slices are given in the shape of 512 × 512. Image rescaling is important to reduce the proposed model’s training time because of fewer parameters and memory usage.

### 2.2. Feature Extraction Using ResNet101-V2 and Inception-V4

Feature extraction is important as it reduces the model’s parameters and computation cost, ultimately enhancing the ICH detection and subtype classification performance. Therefore, the windowing and adjacent slicing-based inputs are passed to the two feature extraction techniques, ResNet101-V2 and Inception-V4. These models extract intensity-based and spatial features in a similar way to enhance the identification of ICH and its subtype. To extract the potential windowed features, this paper proposes a new variant of the ResNet model, known as ResNet101-V2. It depends on the baseline architecture of CNN that extracts high-level features from the given input. Many studies demonstrate the importance of deep learning to capture the hidden complexities efficiently and significantly improve the ICH detection performance [37,38]. The authors Wang et al. in [2,8,39,40] employ deep learning feature extractors such as EfficientNet-B0, DenseNet121, Capsule network, and Visual geometry group (VGG), respectively. Although, with the deep architecture, the gradient vanishes during back-propagation and leads to poor performance, resolved through the skip connections in the ResNet architecture. Therefore, this paper utilizes the pre-trained deep learning architecture of ResNet101-V2 that consists of residual blocks and skip links to efficiently obtain the intensity base features from CT scans. Specifically, it is deep with 101 layers that efficiently find the subtle lesions. The deep architecture of ResNet101-V2 has achieved more excellent performance than the shallow levels architecture such as ResNet50 and ResNet30 Rahman et al. [37]. In this regard, the pre-trained architecture of ResNet101-V2 is employed as a feature extractor to enhance the ICH detection performance, given Keras et al. [41,42].

The spatial features are obtained parallel to the intensity features to follow the real-world diagnostic process of ICH detection. The spatial information in the proposed methodology is obtained through the Inception-V4 model that receives the adjacent slices as input. The prime intention behind using the inception-V4 is the identification of hidden bleeding regions at low computation cost and high performance [43]. It has achieved the highest performance in the classification of ImageNet due to efficient training speed, and fewer model parameters [44]. Likewise to the ResNet101-V2, it is based on the CNN architecture and has both residual skip connections and inception blocks to improve the ICH detection rate. Therefore, in this paper, we use an effective pre-trained feature extractor, Inception-V4, as stated Keras et al. [41,43]. The ResNet101-V2 returns the 2048 intensity-based features, whereas 1536 spatial features are obtained through the Inception-V4. Afterwards, the features provided by both feature extractors are concatenated to form a new input for ICH detection and subtype characterization.

### 2.3. ICH Detection and Subtype Classification

The final detection and five subtype classification of ICH are performed by the LGBM which uses the concatenated windowed and spatial features. The researchers use SVM [11], DT [14], RF [19], and DT-, RF-, SVM-, and MLP-based four machine learning models in [16] to identify and characterize the ICH. However, these machine learning models become expensive for large datasets, leading to overfitting problems. Moreover, these models have slow feature learning mechanisms and poorly detect ICH. Therefore, in this paper, we have used the boosting mechanism known as LGBM, which is light and has fast training. The boosting strategy learns from the mistakes of previous classifiers and optimizes the performance of the upcoming classifier [45]. It significantly enhances the performance of ICH detection and its subtype classification through rapid learning, less memory consumption, and minimization of previous mistakes during the training process.

Moreover, it can efficiently handle a large dataset, which makes it suitable for this study [46]. LGBM follows the leaf wise strategy to solve the hidden complexities compared to other tree-based models, such as DT or extreme gradient boosting (Xgboost) that employ a level-wise approach. Therefore, the leaf wise strategy of LGBM helps find the hidden complexities of ICH and enhance its detection score. LGBM has several important hyperparameters, such as the number of classifiers, depth, and learning rate. These parameters have a significant impact on the ICH detection performance. Thus, the hyperparameters of LGBM are tuned through the grid-search technique.

## 3. Experimental Results

This section describes the experimental results of the proposed methodology to assess its performance for subtype classification and detection of ICH. The proposed solution is compared with the existing benchmark techniques for ICH detection to demonstrate its effectiveness and suitability for real-time application.

The proposed Res-Inc-LGBM solution is developed over the workstation with specifications of Intel core i7-7200, 16GB RAM, and a 1TB hard drive using the Python language. In addition, the TensorFlow and Keras libraries and the graphics processing unit (GPU) of NVIDIA are employed to form and train the deep learning models. The proposed Res-Inc-LGBM is developed and assessed over the dataset of IHDC, which is mentioned in Section 2.1.1. The dataset is divided into two subsets, training and testing, with a ratio of 70 and 30, respectively. In this study, the pre-trained architecture of ResNet101-V2 and Inception-V4 are employed to capture the windowed and spatial features [45].

The proposed architectures are trained using the adaptive moment estimation (Adam) learning technique and weighted binary cross-entropy (WBC) loss function. The weighted loss function is used to deal with the effects of imbalanced data. The weight value is assigned concerning the class importance, such as 1.0, 0.29, 0.22, 0.15, 0.16, and 0.18 for ‘Any’, EDH, IVH, IPH, SDH, and SAH subtypes. The CT scans are reshaped to 224 × 244 and 299 × 299 for forwarding as an input of ResNet101-V2 and Inception-V4, respectively. The feature extractors use the batch size of 16 and learning rate of 1×10-3 to train over 50 iterations where the performance of models is evident. Afterwards, the extracted features are received as input by LGBM for ICH detection and its subtype categorization, which is mentioned in Section 2.3.

### 3.1. Experimental Results of Proposed Solution

To assess the performance of the proposed Res-Inc-LGBM algorithm, several classification-based performance indicators are employed in this study. These indicators are obtained through the confusion matrix that provides four outcomes: true positive (TP), false positive (FP), true negative (TN), and false negative (FN).

These four outcomes provide the basis to form several potential performance indicators that are used in this study, such as F1-measure, precision, sensitivity (also known as recall) and TP rate (TPR), specificity, also identified as TN rate (TNR), area under the precision-recall (AUPR), accuracy, and area under the curve (AUC) Sage et al. [19,46].

**Figure 5:**
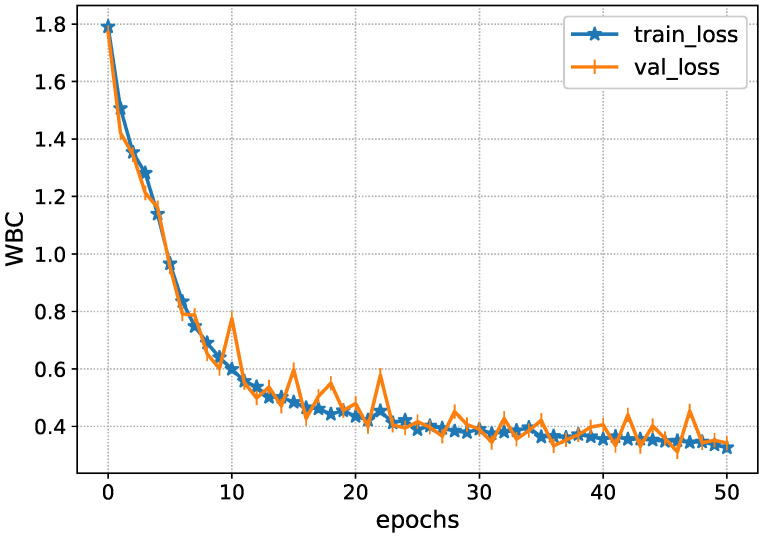
Loss analysis of Inception-V4 during training.

After the preprocessing of data, feature extraction techniques, Inception-V4, and ResNet101-V2, are applied to obtain important features. The Inception-V4 extracts the important elements from the adjacent CT slices. Specifically, it finds the complex bleeding regions through the adjacent CT slices. Figure 5 shows the performance of the Inception-V4 while obtaining the spatial features. It is seen that the deep architecture of Inception-V4 efficiently learns the subtle complexities and minimizes the loss function.

**Figure 6:**
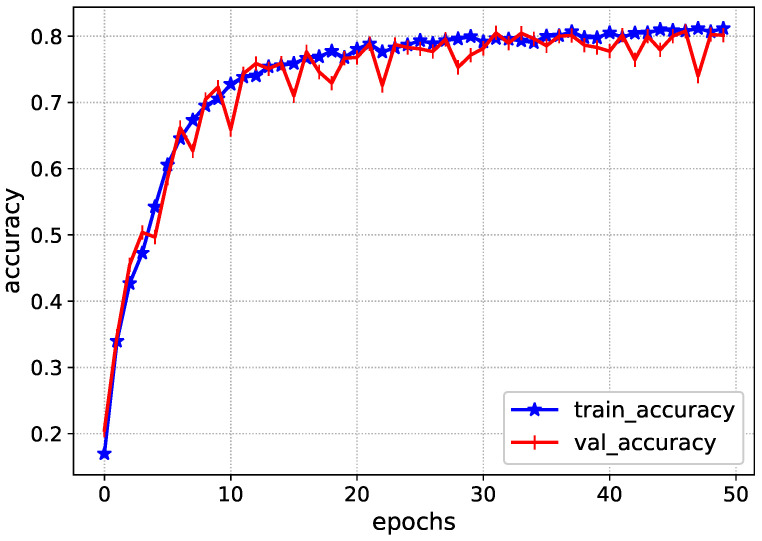
Accuracy of Inception-V4 during training.

The Inception-V4 has uniform performance for both training and validation data, such as the loss values of 0.01. During the validation, the model loss sometimes fluctuates due to the complexity of ICH. Afterwards, the accuracy of the Inception-V4 is shown in Figure 6 during the model training. It achieves an accuracy level of 0.85 during the training, showing the proposed solution’s excellency.

**Figure 7:**
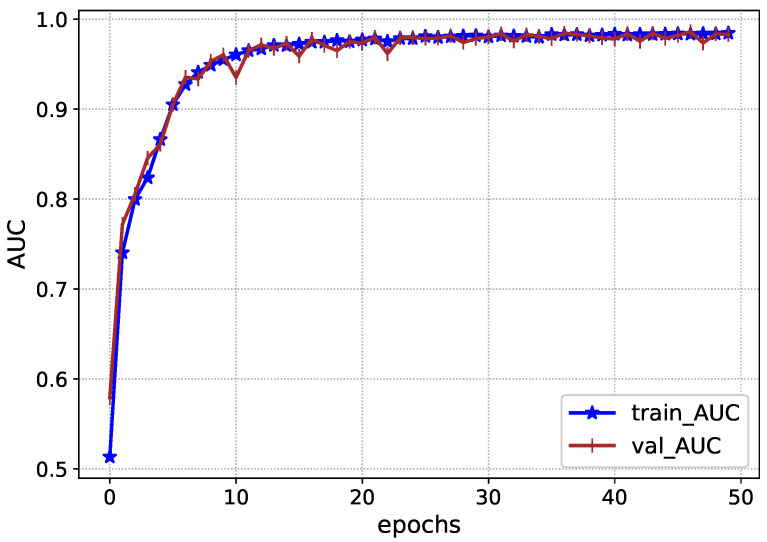
AUC during the training Inception-V4.

**Figure 8:**
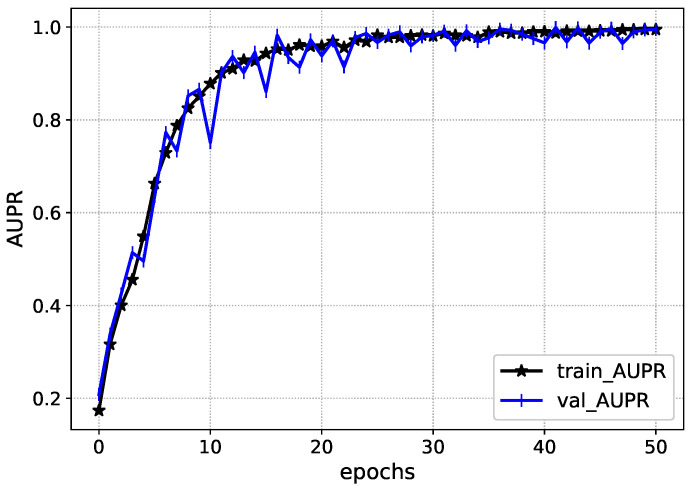
AUPR of Inception-V4 during execution.

The performance of Inception-V4 in terms of AUC and AUPR is shown in Figure 7 and Figure 8 during the model execution, respectively. As it is clear that the model excellently finds the hidden complexities of ICH, which the AUC and PRAU validate. The Inception-V4 feature extractor obtains scores of 0.97 and 0.98 for AUC and PRAU, respectively. These scores are obtained using the training and validation sets. The execution results of Inception-V4 validate that the proposed mechanism has significant potential to enhance ICH detection and its subtype classification.

**Figure 9:**
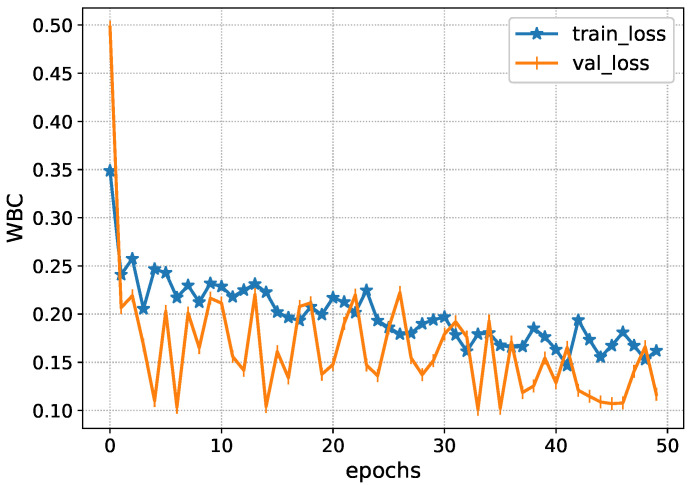
Loss analysis of ResNet101-V2 during training.

**Figure 10:**
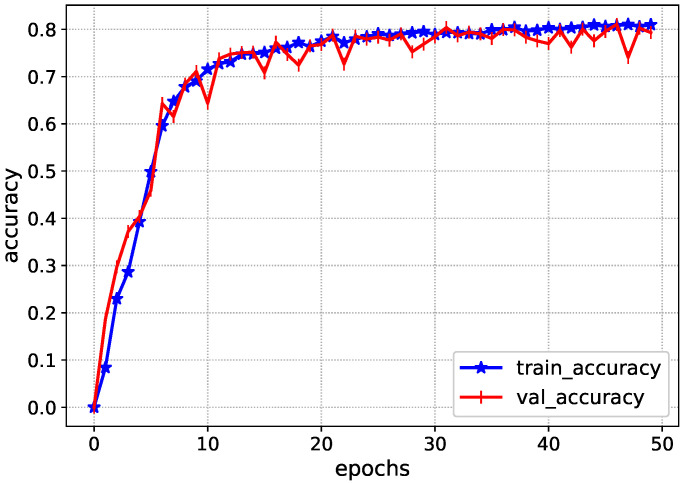
Accuracy of ResNet101-V2 during training.

In the parallel way of spatial features, the intensity-based representations are achieved through another feature extractor, ResNet101-V2. It has the potential to efficiently extract the brain, bone, and subdural tissues based on subtle complexities, as shown in Figure 9. It is depicted that the second model also achieves excellent results for feature extraction on training and validation sets. However, the fluctuations are seen for the validation data due to complexities and similarities between the subtypes of ICH, such as EDH and SDH. Except that, the model efficiently minimizes the loss value and obtains 0.10 for the validation set, as shown in Figure 9. Likewise, the accuracy of the ResNet101-V2 is depicted in Figure 10. The figure shows the accuracy of training and validation sets regarding the extraction of brain, bone, and subdural tissues.

**Figure 11:**
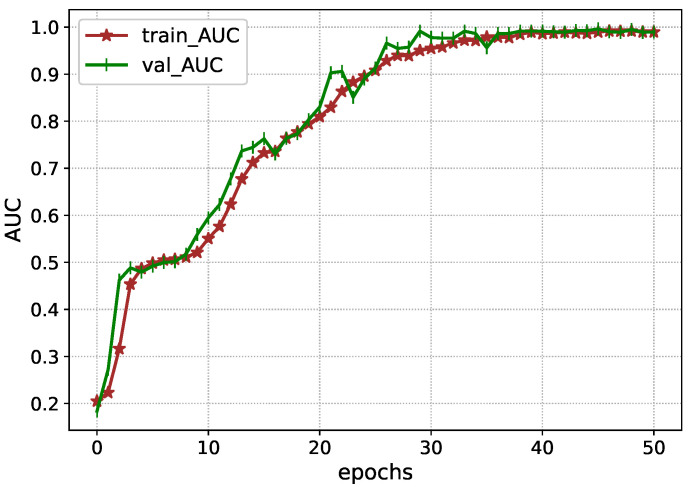
AUC during the training ResNet101-V2.

**Figure 12:**
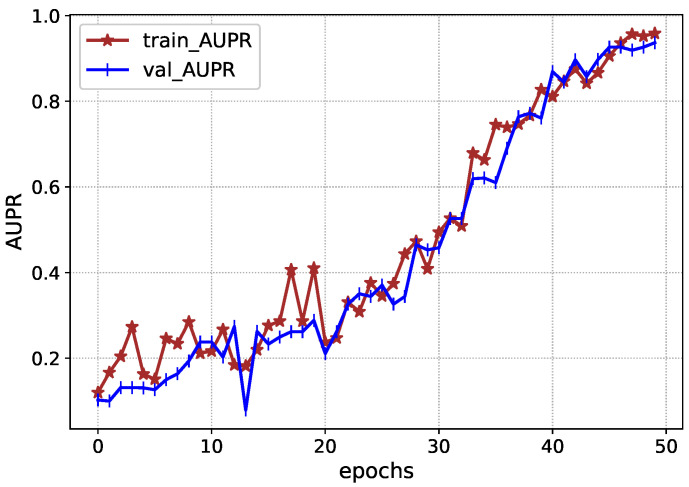
AUPR of ResNet101-V2 during execution.

In contrast to the loss value, the accuracy value gradually increases for both training and validation sets. It is evident that ResNet101-V2 efficiently finds insights from CT scans and improves the accuracy level. In addition, to demonstrate the efficiency of the ResNet101-V2 for the extraction of intensity features, the AUC and AUPR scores are also depicted in Figure 11 and Figure 12. It is shown in Figure 11 that ResNet101-V2 smoothly optimizes the AUC for both training and validation sets. The AUC score of 0.98 is obtained by the model for training and unseen dataset. Likewise, Figure 12 highlights the AUPR-based performance over the model training iterations. The figure depicts that the ResNet101-V2 uniformly improves the AUPR score for the training and testing sets regarding the extraction of intensity features.

**Figure 13:**
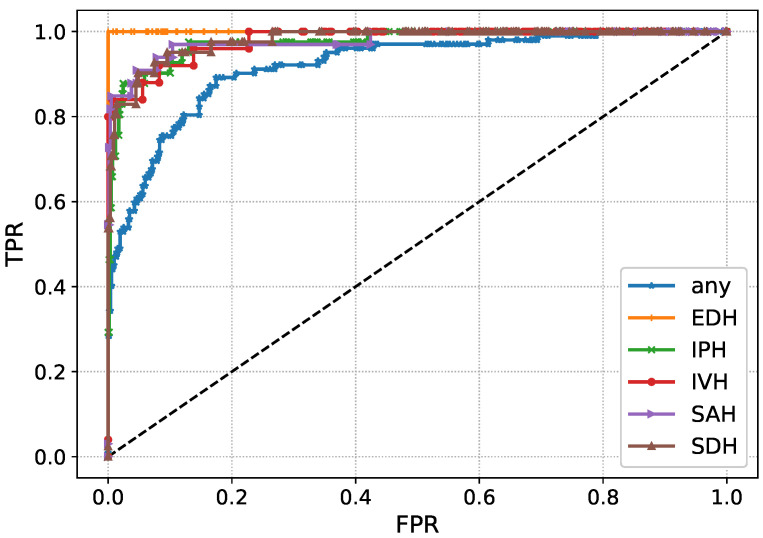
AUC-based performance of the proposed solution.

**Figure 14:**
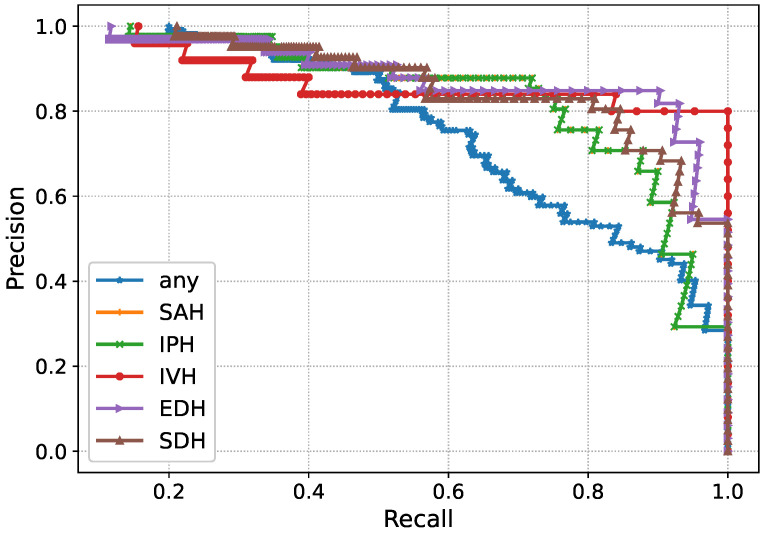
Proposed solution’s performance based on AUPR.

The AUC- and AUPR-based performance of the ResNet101-V2 validates that the employed feature extractor has significant capability to obtain the hidden bleeding regions from intensity-based CT slices. Furthermore, the AUC-based performance of the proposed solution in terms of the receiver operating characteristics (ROC) curve is shown in Figure 13. The ROC curves depict the TPR versus FPR to demonstrate the ICH detection and its subtype classification ability of the model. The high value of the curve states the excellent detection power of the proposed mechanism. In a similar fashion to AUC, the proposed solution’s performance regarding AUPR is depicted in Figure 14 where precision against recall is demonstrated. The curves in the figure show the performance for each subtype of ICH. In general, the feature extraction results of ResNet101-V2 and Inception-V4 validate that these models efficiently the detection and subtype classification of ICH.

To assess the performance of the proposed Res-Inc-LGBM solution for ICH detection and its subtype categorization, Table 2 presents the complete analysis of the results. The table illustrates the performance results for each of the subtypes of ICH. It is seen that the proposed Res-Inc-LGBM achieves 0.985 AUC, 0.954 sensitivity, 0.972 specificities, 0.947 precision, 0.963 F1-score, 0.974 AUPR, and 0.975 accuracies for ICH detection. The proposed solution obtains efficient results for ICH detection due to the efficiency of hybrid deep learning and machine learning techniques. The deep learning features extractors capture the important intensity and spatial features that significantly improve the ICH subtype classification and detection, as stated in Table 2.

Furthermore, the performance results for SDH subtype detection are 0.974 AUC, 0.965 sensitivity, 0.983 specificities, 0.963 precision, 0.972 F1-score, 0.971 AUPR, and 0.987 accuracies. These results show significant improvement in detecting SDH due to its similarity with EDH. Likewise, 0.980, 0.972, 0.963, 0.966, 0.952, 0.964, and 0.975 are obtained for detecting EDH in terms of AUC, sensitivity, specificity, precision, F1-score, AUPR, and accuracy, respectively. The proposed solution secures excellent results for EDH, which is a significant improvement. The traditional studies have fewer detection scores for SDH and EDH due to their hidden subtleties and similarities.

Moreover, the dataset contains less number of EDH cases, which are usually falsely classified by the model. However, the proposed solution has the detection capability that efficiently learns the bleeding regions and reduces the misclassification of EDH. Afterwards, the detection of other ICH subtypes such as SAH, IPH, and IVH are also efficiently covered by the proposed solution with a minimum false detection rate. The detection results of SAH, IPH, and IVH by the Res-Inc-LGBM are also similar to the SDH and EDH, as given in Table 2. In general, the results demonstrate that the proposed solution has significantly improved ICH’s detection and subtype classification.

To validate the effectiveness of ResNet101-V2 for intensity base features and Inception-V4 for spatial, Table 2 presents the results in terms of ResNet101-V2-LGBM (Res-LGBM) and Inception-V4-LGBM (Inc-LGBM). These are the two variants of the proposed Res-Inc-LGBM mechanism to show the importance of intensity and spatial features. In the first variant Res-LGBM, the Inception-V4 is removed from the mechanism to demonstrate the importance of spatial features. Res-LGBM receives windowed CT slices as an input and outputs the intensity features, which are fed as an input to the LGBM for the detection and subtype characterization of ICH. Likewise, the other variant Inc-LGBM highlights the significance of intensity features by removing the Inception-V4 module. The Inc-LGBM model is trained with the adjacent slices as an input and outputs the spatial features.

The LGBM uses spatial features to identify and classify the ICH into subtypes. In terms of ICH detection, the Res-LGBM-based variant gains 0.938 AUC, 0.764 sensitivity, 0.926 specificities, 0.825 precision, 0.843 F1-score, 0.892 AUPR, and 0.926 accuracies. It is seen that the spatial features have a significant impact, such as about 5% to 13% in terms of AUC, sensitivity, F1-score, and AUPR. Likewise, the Inc-LGBM achieves 0.952, 0.803, 0.926, 0.863, 0.875, 0.952, and 0.936 for AUC, sensitivity, specificity, precision, F1-score, AUPR, and accuracy in terms of ICH detection, respectively. The results demonstrate that the intensity-based features also impact the detection score, such as about 3% to 15% regarding AUC, sensitivity, F1-score, and AUPR. It is concluded that the proposed feature extractors have improved the ICH detection and subtype categorization performance due to excellent feature extraction capabilities.

The ICH subtype classification results of Res-LGBM and Inc-LGBM are quite inferior to the results of the proposed Res-Inc-LGBM solution, as given in Table 2. The table states that the performance results of Res-LGBM for EDH are 0.927 AUC, 0.804 sensitivity, 0.896 specificities, 0.883 precision, 0.832 F1-score, 0.844 AUPR, and 0.936 accuracies. In comparison, the Inc-LGBM obtains 0.935 AUC, 0.822 sensitivity, 0.885 specificities, 0.895 precision, 0.875 F1-score, 0.902 AUPR, and 0.945 accuracies for EDH. It is seen that the Inc-LGBM has high AUC, sensitivity, precision, F1-score, AUPR, and accuracy results than the Res-LGBM due to the significance of spatial features and Inception-V4 architecture. Afterwards, for SDH subtype detection, Res-LGBM scores 0.951 AUC, 0.924 sensitivity, 0.986 specificities, 0.905 precision, 0.892 F1-score, 0.931 AUPR, and 0.992 accuracies, whereas Inc-LGBM gains 0.957 AUC, 0.931 sensitivity, 0.963 specificities, 0.962 precision, 0.893 F1-score, 0.945 AUPR, and 0.996 accuracies.

The Inc-LGBM has results superior to those of the Res-LGBM for AUC, sensitivity, F1-score, AUPR, and accuracy in terms of SDH. Specifically, the Inc-LGBM has more excellent results for ICH detection and subtype categorization than the Res-LGBM, as stated in Table 2, although it is clear that the spatial features have more impact on ICH detection performance than the intensity-based features. In the combination of both spatial and windowed features, the proposed Res-Inc-LGBM achieves more excellent results than its two variants, as validated by the performance results in Table 2.

The proposed solution’s generalization ability can be seen in Table 3 by testing it on the CQ500 dataset. The table presents ICH detection and its subcategorization results regarding recall (sensitivity), specificity, F1-score, and accuracy. The performance results clearly state the proposed methodology has excellent generalization ability for ICH detection. Specifically, using the CQ500 dataset, the proposed Res-Inc-LGBM achieves 95.4%, 94.2%, 94.1%, and 95.1% for sensitivity, specificity, F1-score, and accuracy, respectively. Therefore, the proposed solution efficiently handles the complex task of ICH detection using the unseen dataset.

### 3.2. Performance Comparison with Benchmarks

Here, we demonstrate the performance of the proposed solution against other state-of-the-art techniques, Wang et al. [2,8,17,19,26,27] for ICH detection, and subtype classification. The authors in [2] achieve 0.964 AUC, 0.944 specificities, and 0.887 sensitivity for ICH detection compared to the proposed model that scores 0.985 AUC, 0.972 specificities, and 0.954 sensitivity. The proposed solution achieves more excellent results due to its efficient strategy of LGBM with rapid training. Afterwards, the proposed Res-Inc-LGBM compares with the approach presented in [8] where the benchmark model covers 0.943 for AUC and 0.859 for sensitivity in ICH detection, whereas the proposed mechanism achieves 0.985 AUC and 0.954 sensitivity. Furthermore, the approach presented in [8] achieves 0.792 sensitivity, 0.823 F1-score, and 0.986 accuracies for EDH. In contrast, the proposed Res-Inc-LGBM model obtains 0.972 sensitivity, 0.952 F1-score, and 0.975 accuracy to detect the EDH, which is quite a complex subtype of ICH due to its similarity with other subtypes of ICH.

The researchers Keshavamurthy et al. [17] achieve an accuracy level of 0.905, a sensitivity of 0.936, 0.964 AUC, and 0.882 specificities to detect the brain injury using CNN and linear SVM model. The proposed technique finds more excellent results, such as an accuracy level of 0.975, sensitivity of 0.954, 0.985 AUC, and 0.972 specificities. Another similar mechanism is proposed in [19] that gets a score of 0.902 for AUC, 0.894 for specificity, 0.897 for accuracy, and 0.901 for sensitivity to identify the ICH. On the other hand, the Res-Inc-LGBM has more excellent results for ICH detection and subtype categorization, as mentioned in Table 2. For instance, the approach based on [19] achieves 0.727 for identifying EDH subtype in terms of F1-score, whereas the Res-Inc-LGBM covers 0.952 F1-score. Bruja et al. [26] present a CNN- and LSTM-based model that achieves a score of 0.865 for sensitivity, 0.957 for specificity, 0.972 for AUC, and 0.96 for accuracy regarding the ICH detection. In contrast, the proposed model has more excellent results for ICH detection than the [26], as given in Table 2. Another study [27] also implements the same mechanism of CNN and LSTM that achieves 0.956 for AUC versus the proposed solution that covers the AUC score of 0.985 for ICH detection.

The proposed Res-Inc-LGBM overcomes the shortcomings of standard techniques regarding subtype classification and detection of ICH. The main reason behind this is the efficiency in feature extraction and detection through the LGBM, which is faster and more efficient for the identification and subtype classification of ICH. Therefore, the proposed mechanism is an efficient and suitable choice for ICH detection and its subtype categorization.

## 4. Conclusions

In this paper, ICH detection and its subtype classification are improved by presenting the new solution, known as Res-Inc-LGBM, using the dataset of IHDC. The proposed Res-Inc-LGBM mechanism uses two deep learning feature extractors, ResNet101-V2 and Inception-V4, to find the significant spatial and intensity features. Afterwards, the deep learning feature extractors are combined with the more efficient machine learning boosting technique, LGBM, that uses the extracted features as input and performs the subtype categorization and detection of ICH. The complete methodology is evaluated by extensive experimentation to show its excellent performance and suitability for real-time ICH detection.

The experiment results of the proposed Res-Inc-LGBM for ICH detection show that the discriminative capability of the model was excellent in terms of AUC, sensitivity, specificity, precision, F1-score, AUPR, and accuracy, respectively. Moreover, the proposed Res-Inc-LGBM is compared against the state-of-the-art techniques regarding ICH detection and its subtype characterization. The proposed model overcomes the performance of existing techniques for ICH detection, which shows its excellent detection capability and suitability for real-world problems. The proposed solution is developed and assessed over the dataset of IHDC. Therefore, different datasets will be used further to enhance the ICH subtype classification and detection performance.

## Figures and Tables

**Table 1:** Detailed information about the dataset.

Hemorrhage	RSNA Instances		CQ500 Scans
Any	0	11,399	205
	1	1934	
IVH	0	12,881	28
	1	452	
SAH	0	12,708	60
	1	625	
EDH	0	13,279	13
	1	55	
SDH	0	12,488	53
	1	845	
IPH	0	12,666	134
	1	668	

**Table 2:** ICH detection and subtype classification results of the proposed solution using the IHDC dataset.

Subtypes	Model	AUC	Sensitivity	Specificity	Precision	F1-Score	AUPR	Accuracy
any	Res-Inc-LGBM	0.985	0.954	0.972	0.947	0.963	0.974	0.975
	Res-LGBM	0.938	0.764	0.926	0.825	0.843	0.892	0.926
	Inc-LGBM	0.952	0.803	0.926	0.863	0.875	0.952	0.936
SDH	Res-Inc-LGBM	0.974	0.965	0.983	0.963	0.972	0.971	0.987
	Res-LGBM	0.951	0.924	0.986	0.905	0.892	0.931	0.992
	Inc-LGBM	0.957	0.931	0.963	0.962	0.893	0.945	0.996
SAH	Res-Inc-LGBM	0.976	0.962	0.973	0.964	0.963	0.972	0.985
	Res-LGBM	0.967	0.944	0.973	0.925	0.912	0.921	0.972
	Inc-LGBM	0.975	0.901	0.982	0.975	0.932	0.952	0.991
EDH	Res-Inc-LGBM	0.980	0.972	0.963	0.966	0.952	0.964	0.975
	Res-LGBM	0.927	0.804	0.896	0.883	0.832	0.844	0.936
	Inc-LGBM	0.935	0.822	0.885	0.895	0.875	0.902	0.945
IPH	Res-Inc-LGBM	0.979	0.968	0.982	0.973	0.972	0.965	0.987
	Res-LGBM	0.956	0.923	0.951	0.916	0.911	0.903	0.954
	Inc-LGBM	0.963	0.935	0.966	0.953	0.932	0.936	0.967
IVH	Res-Inc-LGBM	0.977	0.962	0.973	0.972	0.963	0.968	0.985
	Res-LGBM	0.953	0.934	0.962	0.943	0.932	0.922	0.963
	Inc-LGBM	0.967	0.954	0.973	0.967	0.959	0.952	0.974

**Table 3:** Evaluation results of Res-Inc-LGBM for ICH detection using CQ500 dataset.

ICH	Recall	Specificity	F1-Score	Accuracy
any	0.952	0.962	0.951	0.971
SDH	0.951	0.953	0.950	0.962
SAH	0.943	0.951	0.946	0.960
EDH	0.954	0.942	0.941	0.951
IPH	0.941	0.935	0.942	0.963
IVH	0.934	0.952	0.937	0.961

## Data Availability

Data will be available upon reasonable request to authors.
